# Severe Sinus Bradycardia in Anorexia Nervosa: A Case Report and Focused Review of Cardiovascular Complications

**DOI:** 10.7759/cureus.73458

**Published:** 2024-11-11

**Authors:** Jamal A Allam, Rayan Abou Zeid

**Affiliations:** 1 Cardiology, Lebanese University Faculty of Medicine, Beirut, LBN; 2 Cardiology, Lebanese University, Beirut, LBN

**Keywords:** anorexia nervosa, bradycardia, cardiovascular complications, electrolyte imbalance, left ventricular mass, malnutrition

## Abstract

Anorexia nervosa (AN) is a severe psychiatric disorder characterized by restricted energy intake, intense fear of weight gain, and body image disturbances. It predominantly affects adolescent females, with rising prevalence, especially during the COVID-19 pandemic. AN leads to multiple medical complications, including cardiovascular issues such as bradycardia, which may result from malnutrition, electrolyte disturbances, and myocardial atrophy. We report a case of a 19-year-old female patient with a three-month history of weight loss, resulting in a weight of 38 kg and a body mass index of 14 kg/m², indicative of severe malnutrition. She presented with a fluctuating appetite, fatigue, muscle pain, mood changes, and sleep disturbances. Clinical evaluation revealed severe sinus bradycardia (30-40 bpm) and a small heart volume, with reduced stroke volume (<45 mL/beat) and cardiac output (<3.5 L/min). Electrolyte analysis showed mildly reduced magnesium, and inflammatory markers showcasing mild elevation of erythrocyte sedimentation rate. Additional psychiatric findings, including obsessive weight monitoring and self-induced vomiting, led to a diagnosis of AN. This report underscores the importance of recognizing bradycardia as a significant cardiovascular manifestation of AN. Early psychiatric intervention and multidisciplinary management are crucial to address malnutrition, electrolyte imbalances, and cardiac dysfunction to prevent further complications and improve patient outcomes.

## Introduction

Eating disorders are present in approximately 1%-2% of adolescent girls and 0.5% of boys, including conditions like anorexia nervosa (AN), bulimia, and binge eating disorder. The prevalence of these conditions has been rising, especially among teenagers, with mental health comorbidities and a heightened risk of suicide as key contributing factors, which vary across the different subtypes [1,2].

AN, which involves severe dietary restriction, significant weight loss, an intense fear of gaining weight, and body image distortions, typically begins in adolescence and primarily affects females. The incidence of AN has seen an upward trend, particularly during the COVID-19 pandemic, leading to malnutrition that causes serious medical complications and a high mortality rate. Treatment options are limited, and outcomes are often unsatisfactory [3,4].

AN and related eating disorders have a decade-long mortality rate of 5.6%, with deaths often due to suicide or cardiac complications. Common issues linked to AN include stunted growth, delayed puberty, reduced bone density, mental health conditions, substance abuse, and self-harm [5].

Cardiovascular complications are frequent in eating disorders, manifesting as hypotension, bradycardia, and myocardial atrophy. These issues are associated with hypovolemia, decreased cardiac output, and increased peripheral vascular resistance, leading to higher morbidity and an elevated risk of premature death [6,7].

This study aims to evaluate the cardiovascular complications associated with AN, with specific emphasis on severe sinus bradycardia and its impact on patient health.

## Case presentation

A 19-year-old unmarried female patient with no history of smoking or chronic illnesses presented to the Internal Medicine Department at the Central Military Hospital, Beirut, Lebanon, with complaints of a three-month history of severe weight loss, having lost approximately 18 kg from a previous weight of 56 kg to her current weight of 38 kg and body mass index (BMI) of 14 kg/m². This was accompanied by a fluctuating appetite, marked fatigue, generalized weakness, and widespread muscle pain. She also reported mood disturbances, including persistent sadness, heightened stress, sleep disturbances, and a sedentary lifestyle. The patient denied symptoms such as fever, chills, cough, sweating, diarrhea, abdominal pain, or breathing difficulties. There was no recent travel or known exposure to infectious diseases. Her family history was unremarkable for similar medical or psychiatric conditions.

This report was approved by the Institutional Review Board (IRB) of Central Military Hospital, Beirut, Lebanon (IRB number: 30/2024). The patient provided written informed consent for the publication of this case report.

Upon physical examination, the patient was found to have stable vital signs, except for pronounced bradycardia, with a heart rate ranging from 30 to 40 beats per minute. She appeared ill, lethargic, anorexic, and pale, with a depressed mood. Despite her generalized weakness, there were no signs of skin bruising or palpable masses on examination. Auscultation revealed normal heart sounds, and her chest was clear to auscultation. Mild tenderness was noted upon palpation of muscles and skin, though no localized pain or masses were identified.

During the clinical evaluation, additional information provided by the patient's mother raised several critical psychiatric red flags. Her mother revealed that the patient had experienced significant weight gain during childhood but had drastically lost weight over the preceding three months. She also described the patient's frequent episodes of self-induced vomiting, obsessive mirror-checking, and daily weight measurement. These behaviors, coupled with the patient's mood disturbances, fluctuating appetite, and fatigue, raised a strong suspicion of AN. Recognizing the psychiatric nature of her disorder, the medical team promptly referred the patient to a psychiatric team for further assessment and management.

A comprehensive initial work-up was performed to investigate the causes of her malnutrition and bradycardia. Hemoglobin levels and thyroid function (thyroid-stimulating hormone) were within normal limits, though mild iron deficiency was identified, while vitamin B12 levels remained normal. Inflammatory markers indicated a mildly elevated erythrocyte sedimentation rate with a normal C-reactive protein level. Serum creatinine was decreased, but electrolyte analysis revealed only a slight reduction in magnesium levels. Cardiac enzymes, including creatine kinase (CK) and CK-MB, were normal, and a chest X-ray (CXR) showed no abnormalities.

**Figure 1:**
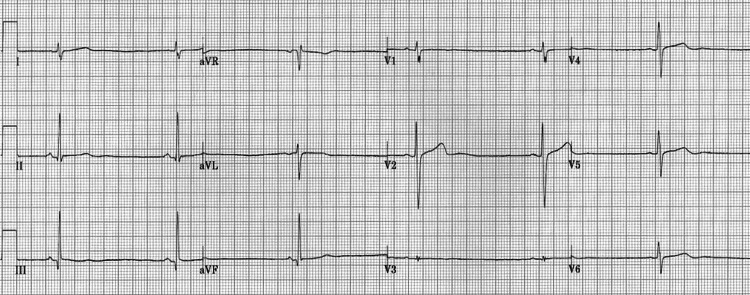
The EKG of the patient showed a sinus bradycardia pattern aVR: augmented vector right; aVL: augmented vector left; aVF: augmented vector foot; EKG: electrocardiogram

**Figure 2:**
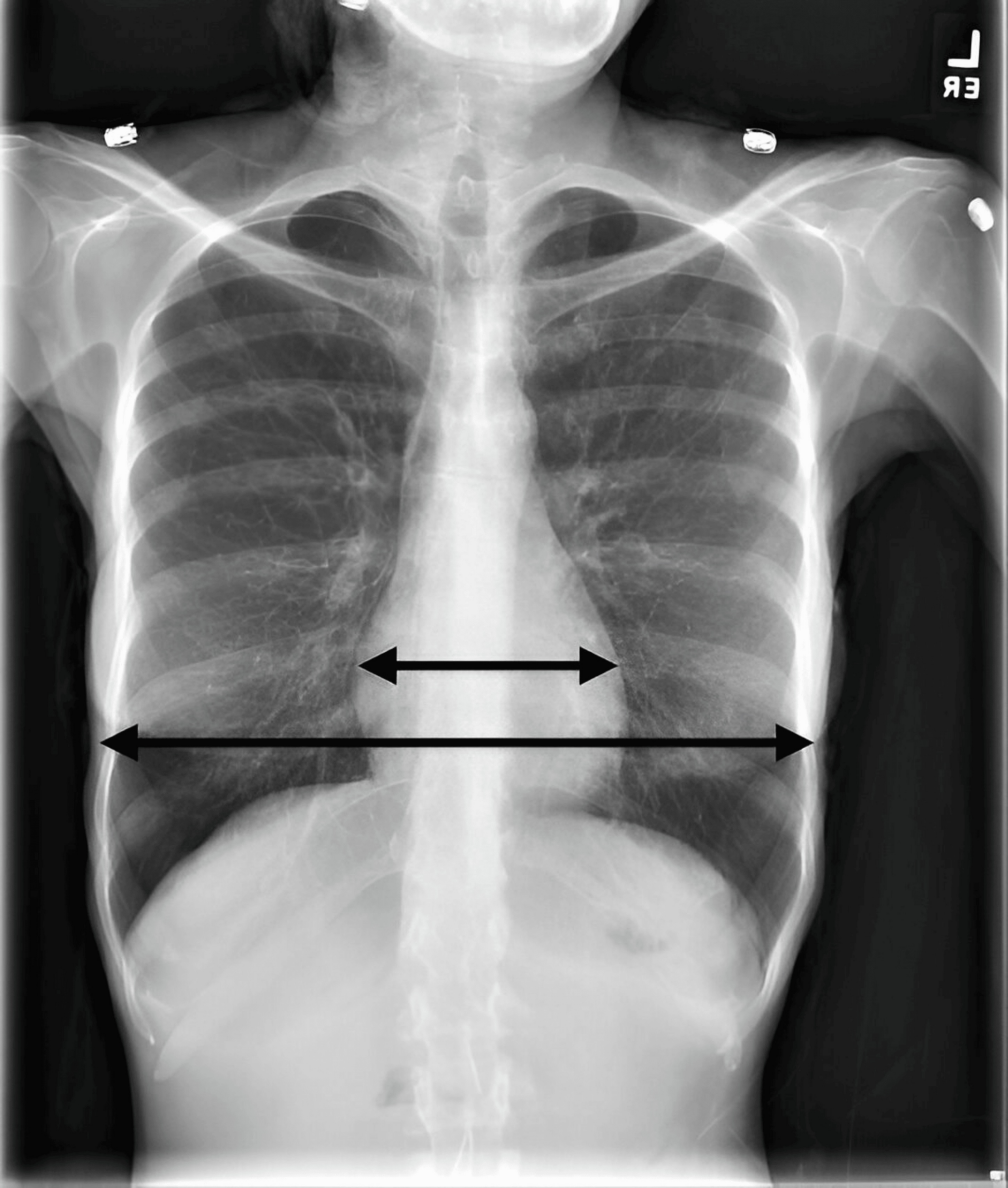
The CXR taken as part of the admission protocol showing the patient's small heart shadow and decreased cardiothoracic ratio (note the difference between the small and large arrows) CXR: chest X-ray

An electrocardiogram (ECG) demonstrated severe sinus bradycardia (Figure 1), with a heart rate of 30-40 bpm, significantly lower than the normal range of 60-100 bpm for a young adult. A transthoracic echocardiogram revealed a small heart volume, similar to changes seen in elderly patients, with a stroke volume of less than 45 mL/beat (normal range: 60-100 mL/beat) and a cardiac output of less than 3.5 L/minute (normal range: 4.5-6.0 L/minute). The reduced heart size was confirmed by the significantly decreased cardiothoracic ratio seen on imaging (Figure 2), which shows a small heart shadow on the CXR with a notable difference between the small and large arrows indicating the reduced cardiothoracic ratio. Despite these findings, no structural heart abnormalities were identified.

Final diagnosis and follow-up

The final diagnosis for the patient was AN with severe sinus bradycardia, attributed to malnutrition. The patient was admitted to a multidisciplinary care team consisting of psychiatric, medical, and nutritional specialists. A nutritional rehabilitation plan was initiated, focusing on gradual refeeding and electrolyte correction. Psychiatric evaluation confirmed the diagnosis of AN, and therapy was started to address her distorted body image and obsessive behaviors.

The patient's bradycardia and cardiac output gradually improved over the course of treatment. During a two-month follow-up, her heart rate increased to 50-60 bpm, and she reported improved energy levels, mood stabilization, and reduced muscle pain. Ongoing psychiatric and nutritional support was arranged as part of her long-term management plan, with continued monitoring of her cardiac function to prevent any further complications.

## Discussion

AN has the highest mortality rate of any psychiatric disorder [8]. It is defined by a severe restriction in caloric intake relative to energy expenditure. Individuals with AN often experience an overwhelming fear of gaining weight and a distorted perception of their body, leading to an unawareness of the gravity of their low body weight. Given its relatively low lifetime prevalence of 0.9% in women and 0.3% in men [9], AN is rarely encountered by general cardiologists, electrophysiologists, and primary care physicians. However, it presents a significant risk for early mortality [10,11].

AN is a complex disorder with both psychological and physiological components, contributing to high rates of psychiatric comorbidities. Lifetime prevalence of comorbid conditions includes depression (15%-60%), anxiety disorders (20%-60%), and substance abuse (12%-21%) [12]. In this case, malnutrition and electrolyte imbalances, notably a slight reduction in magnesium levels, had a detrimental impact on the patient’s health, leading to muscle pain and overall weakness.

In AN, the effects of malnutrition include muscle wasting, decreased protein synthesis, and subsequent weakness, fatigue, and pain. Electrolyte disturbances, such as hypokalemia and hypomagnesemia, disrupt nerve function and muscle contractions, causing cramps, spasms, and myalgia [13].

This study highlights that the cardiovascular assessment revealed marked sinus bradycardia (30-40 bpm), decreased cardiac capacity, and a reduction in both stroke volume (<45 mL) and cardiac output (<3.5 L/minute), illustrating the impact of malnutrition on heart size and function, commonly observed in AN. Consistent with our findings, several other studies have confirmed that bradycardia is a frequent occurrence in individuals with AN [14-16].

In one retrospective study, 16 of 20 patients diagnosed with AN presented with sinus bradycardia [17]. Another study involving 38 adolescent patients demonstrated a 68% prevalence of bradycardia. Interestingly, patients with a longer duration of illness were less likely to exhibit bradycardia, and BMI was found to be a predictor of a lower heart rate. Additionally, heart rate was shown to be a predictor of spinal bone mineral density, indicating a more severe degree of malnutrition [18].

Another study involving 171 adolescents with AN revealed that greater total and recent weight loss was linked to a lower heart rate nadir, and these characteristics were strongly associated with a higher incidence of bradycardia [19]. In a study of 33 adolescent males with eating disorders, the average heart rate was 58.7 bpm, with a mean orthostatic heart rate change of 22 beats/minute. However, this study focused solely on a male population and did not compare the results with those of female patients or control groups [20].

Cardiovascular complications are common in individuals with AN, with sinus bradycardia being the predominant cardiovascular manifestation and the most frequent arrhythmia in this population [14]. It is crucial to recognize its significance in this clinical setting, as it can be associated with sudden death, especially when other arrhythmias or ECG abnormalities, such as prolonged corrected QT interval from electrolyte imbalances (e.g., hypokalemia, hypomagnesemia, and hypophosphatemia), or congenital conditions like long QT syndrome, are present [15].

Bradycardia in AN is believed to stem from a physiological adaptation, primarily due to enhanced vagal tone and reduced energy metabolism resulting from insufficient caloric intake [16]. It has been suggested that bradycardia arises from structural alterations in the heart, including a reduction in left ventricular muscle mass, as a consequence of malnutrition [21]. In patients with heart atrophy, it has been proposed that bradycardia may act as a compensatory mechanism to avert heart failure. Vázquez et al. observed decreased glycogen content in myocardial cells along with cellular shrinkage, which may play a role in the development of bradycardia [22].

There is evidence indicating that the bradycardia associated with AN is reversible. With appropriate treatment, clinical recovery, and weight gain, heart rate improves at rest and during physical activity. However, in some individuals undergoing significant weight loss, bradycardia may result from dysfunction in the cardiac conduction system, potentially requiring permanent pacemaker implantation [23-25]. Patients with AN who are admitted with a heart rate below 35 bpm should undergo continuous monitoring, and the use of inotropic agents is contraindicated due to the risk of exacerbating ventricular arrhythmias [14].

## Conclusions

This case underscores the importance of recognizing bradycardia as a key cardiovascular manifestation of AN, often resulting from malnutrition and adaptive hypometabolism. Multidisciplinary care, including nutritional support, cardiac monitoring, and psychiatric intervention, is essential to improving patient outcomes and preventing severe complications. Early recognition and targeted treatment can aid in reversing cardiovascular effects and stabilizing health.
